# Dapagliflozin reduces thrombin generation and platelet activation: implications for cardiovascular risk reduction in type 2 diabetes mellitus

**DOI:** 10.1007/s00125-021-05498-0

**Published:** 2021-06-16

**Authors:** Christina Kohlmorgen, Stephen Gerfer, Kathrin Feldmann, Sören Twarock, Sonja Hartwig, Stefan Lehr, Meike Klier, Irena Krüger, Carolin Helten, Petra Keul, Sabine Kahl, Amin Polzin, Margitta Elvers, Ulrich Flögel, Malte Kelm, Bodo Levkau, Michael Roden, Jens W. Fischer, Maria Grandoch

**Affiliations:** 1 Institute of Pharmacology and Clinical Pharmacology, Medical Faculty and University Hospital of Düsseldorf, Heinrich-Heine University Düsseldorf, Düsseldorf, Germany; 2 Cardiovascular Research Institute Düsseldorf (CARID), Medical Faculty and University Hospital of Düsseldorf, Heinrich-Heine University Düsseldorf, Düsseldorf, Germany; 3 Department of Cardiothoracic Surgery, Heart Center, University Hospital of Cologne, University of Cologne, Cologne, Germany; 4 Institute for Clinical Biochemistry and Pathobiochemistry, German Diabetes Center, Leibniz Center for Diabetes Research at the Heinrich-Heine University Düsseldorf, Düsseldorf, Germany; 5 German Center for Diabetes Research (DZD), München-Neuherberg, Germany; 6 Division of Vascular and Endovascular Surgery, Experimental Vascular Medicine, Heinrich-Heine University Medical Center, Medical Faculty and University Hospital of Düsseldorf, Heinrich-Heine University Düsseldorf, Düsseldorf, Germany; 7 Division of Cardiology, Pulmonology, and Vascular Medicine Medical Faculty, University Hospital of Düsseldorf, Heinrich-Heine University Düsseldorf, Düsseldorf, Germany; 8 Institute for Molecular Medicine III and University Hospital Düsseldorf, Heinrich-Heine University Düsseldorf, Düsseldorf, Germany; 9 Institute for Clinical Diabetology, German Diabetes Center, Leibniz Center for Diabetes Research at Heinrich-Heine University Düsseldorf, Düsseldorf, Germany; 10 Experimental Cardiovascular Imaging, Institute of Molecular Cardiology, Medical Faculty and University Hospital of Düsseldorf, Heinrich-Heine University Düsseldorf, Düsseldorf, Germany; 11 Department of Endocrinology and Diabetology, Medical Faculty and University Hospital of Düsseldorf, Heinrich-Heine University Düsseldorf, Düsseldorf, Germany

**Keywords:** Atherosclerosis, Cardiovascular, Dapagliflozin, HDL-cholesterol, Heart failure, Platelets, P-Selectin (CD62P), Sodium–glucose cotransporter 2 (SGLT2) inhibitors, Thrombin

## Abstract

**Aims/hypothesis:**

People with diabetes have an increased cardiovascular risk with an accelerated development of atherosclerosis and an elevated mortality rate after myocardial infarction. Therefore, cardioprotective effects of glucose-lowering therapies are of major importance for the pharmacotherapy of individuals with type 2 diabetes. For sodium–glucose cotransporter 2 inhibitors (SGLT2is), in addition to a reduction in blood glucose, beneficial effects on atherosclerosis, obesity, renal function and blood pressure have been observed. Recent results showed a reduced risk of worsening heart failure and cardiovascular deaths under dapagliflozin treatment irrespective of the diabetic state. However, the underlying mechanisms are yet unknown. Platelets are known drivers of atherosclerosis and atherothrombosis and disturbed platelet activation has also been suggested to occur in type 2 diabetes. Therefore, the present study investigates the impact of the SGLT2i dapagliflozin on the interplay between platelets and inflammation in atherogenesis.

**Methods:**

Male, 8-week-old LDL-receptor-deficient *(Ldlr*^*−/−*^*)* mice received a high-fat, high-sucrose diabetogenic diet supplemented without (control) or with dapagliflozin (5 mg/kg body weight per day) for two time periods: 8 and 25 weeks. In a first translational approach, eight healthy volunteers received 10 mg dapagliflozin/day for 4 weeks.

**Results:**

Dapagliflozin treatment ameliorated atherosclerotic lesion development, reduced circulating platelet–leucocyte aggregates (glycoprotein [GP]Ib^+^CD45^+^: 29.40 ± 5.94 vs 17.00 ± 5.69 cells, *p* < 0.01; GPIb^+^lymphocyte antigen 6 complex, locus G^+^ (Ly6G): 8.00 ± 2.45 vs 4.33 ± 1.75 cells, *p* < 0.05) and decreased aortic macrophage infiltration (1.31 ± 0.62 vs 0.70 ± 0.58 ×10^3^ cells/aorta, *p* < 0.01). Deeper analysis revealed that dapagliflozin decreased activated CD62P-positive platelets in *Ldlr*^*−/−*^ mice fed a diabetogenic diet (3.78 ± 1.20% vs 2.83 ± 1.06%, *p* < 0.01) without affecting bleeding time (85.29 ± 37.27 vs 89.25 ± 16.26 s, *p* = 0.78). While blood glucose was only moderately affected, dapagliflozin further reduced endogenous thrombin generation (581.4 ± 194.6 nmol/l × min) × 10^−9^ thrombin vs 254.1 ± 106.4 (nmol/l × min) × 10^−9^ thrombin), thereby decreasing one of the most important platelet activators. We observed a direct inhibitory effect of dapagliflozin on isolated platelets. In addition, dapagliflozin increased HDL-cholesterol levels. Importantly, higher HDL-cholesterol levels (1.70 ± 0.58 vs 3.15 ± 1.67 mmol/l, *p* < 0.01) likely contribute to dapagliflozin-mediated inhibition of platelet activation and thrombin generation. Accordingly, in line with the results in mice, treatment with dapagliflozin lowered CD62P-positive platelet counts in humans after stimulation by collagen-related peptide (CRP; 88.13 ± 5.37% of platelets vs 77.59 ± 10.70%, *p* < 0.05) or thrombin receptor activator peptide-6 (TRAP-6; 44.23 ± 15.54% vs 28.96 ± 11.41%, *p* < 0.01) without affecting haemostasis.

**Conclusions/interpretation:**

We demonstrate that dapagliflozin-mediated atheroprotection in mice is driven by elevated HDL-cholesterol and ameliorated thrombin–platelet-mediated inflammation without interfering with haemostasis. This glucose-independent mechanism likely contributes to dapagliflozin’s beneficial cardiovascular risk profile.

**Graphical abstract:**

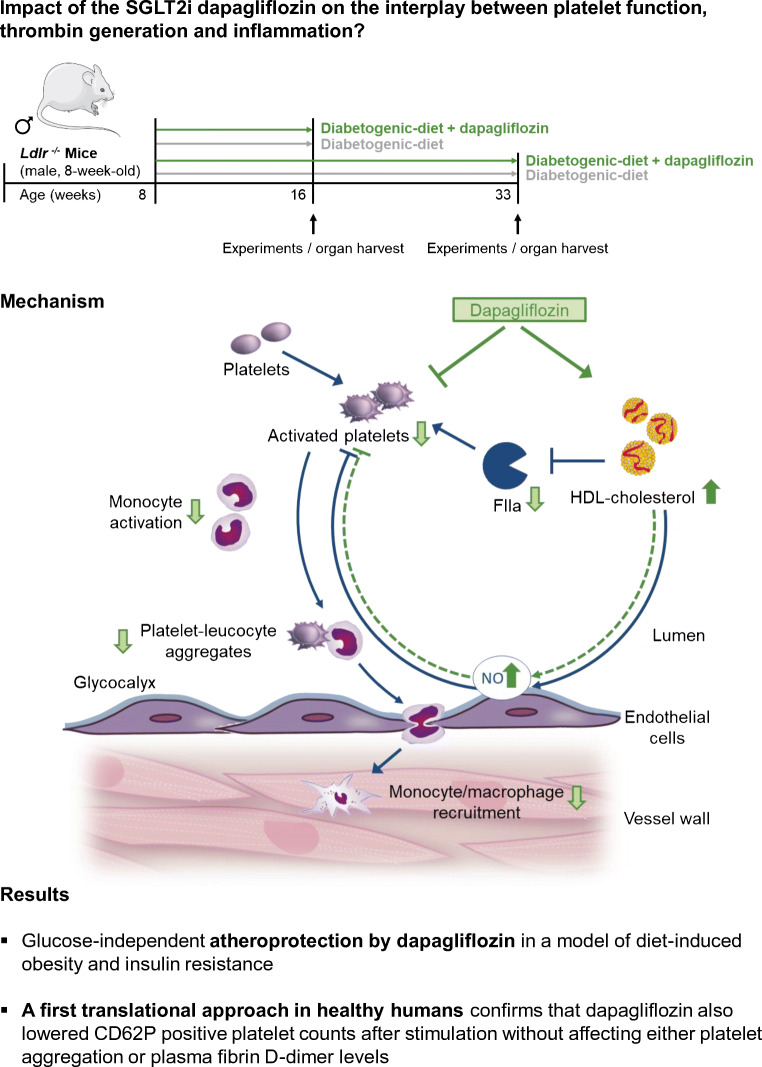

**Supplementary Information:**

The online version of this article (10.1007/s00125-021-05498-0) contains peer-reviewed but unedited supplementary material.

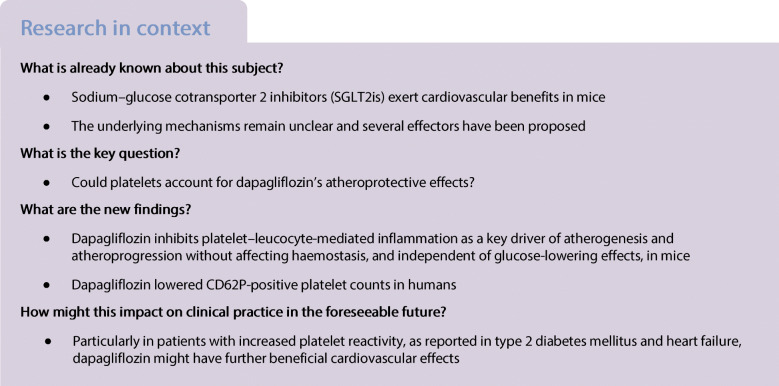



## Introduction

Sodium–glucose cotransporter 2 inhibitors (SGLT2is) have recently been identified as novel therapies in the treatment of diabetes [1]. SGLT2is provide an insulin-independent mechanism for lowering elevated blood glucose in people with type 2 diabetes. Located in the proximal tubule, SGLT2 mediates the majority of renal glucose reabsorption. Inhibiting SGLT2 thus enhances urinary glucose excretion and significantly lowers HbA_1c_ levels in diabetic patients [2–4]. There is a strong clinical need to reduce cardiovascular risk in people with type 2 diabetes because, due to its micro- and macrovascular complications, diabetes accelerates atheroprogression, thereby increasing cardiovascular morbidity and mortality in individuals with type 2 diabetes compared with those without diabetes [5, 6]. Patients with type 2 diabetes have a smaller coronary artery lumen, faster progression of atherosclerosis with higher atherosclerotic plaque burden and a higher atheroma volume. This promotes myocardial infarction or stroke and, even after surviving, the risk of reinfarction and heart failure is high [7, 8]. Accordingly, the cardiovascular effects of new diabetes therapies merit scrutiny. Previous studies show that traditional glucose-lowering compounds such as sulfonylureas, thiazolidinediones and insulin either have no effect on cardiovascular events or actually increase mortality [9, 10]. For the newer class of oral glucose-lowering medications, such as dipeptidyl-peptidase-4 inhibitors and glucagon-like-peptide-1 receptor agonists, a neutral to positive cardiovascular risk profile has been shown in people with type 2 diabetes [6]. Importantly, it should be emphasised that SGLT2is are the first class of glucose-lowering agents with a favourable impact on cardiovascular risk and a risk reduction for heart failure [6]. The DECLARE–TIMI 58 trial confirmed lower cardiovascular death rate and fewer hospitalisations for heart failure in patients with dapagliflozin vs placebo [11].

However, despite these positive findings, the underlying mechanisms still remain unclear and several mechanisms have been discussed. For example, among various other suggested mechanisms, a reduction in blood pressure by SGLT2is has been proposed (probably due to an inhibition of sodium reabsorption), as well as anti-inflammatory effects by targeting the nucleotide-binding oligomerisation domain, leucine-rich repeat, and pyrin domain-containing 3 (NLRP3) inflammasome and an improvement in vascular function by beneficial actions on endothelial cells and mitochondrial function.

This study explores how inhibiting SGLT2 with dapagliflozin affects platelet function in LDL-deficient (*Ldlr*^*−/−*^) mice on a diabetogenic diet, a well-established model of diet-induced obesity and glucose intolerance that closely resembles the situation in humans [12, 13].

In this context conflicting results have been reported [14, 15], but nevertheless this topic is of specific importance for people with diabetes: patients with poorly controlled diabetes have increased platelet-dependent thrombin generation and higher plasma levels of important markers of coagulation [16–18]. Elevated coagulation and platelet activation are known drivers of atherogenesis and atheroprogression [19], leading to cardiovascular events such as myocardial infarction and heart failure. Therefore, agents such as SGLT2is that may also influence platelet activation and thereby atherothrombosis might be a promising strategy in the prevention of cardiovascular events in diabetes; nonetheless, effects of SGLT2is on these parameters have not yet been investigated systematically.

## Methods

### Animal experiments

#### Mouse model

**Fig. 1:**
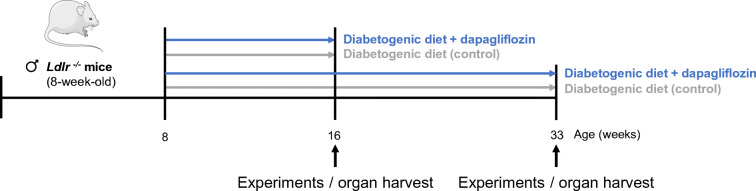
Experimental setting. Male *Ldlr*^*−/−*^ mice were fed DD supplemented without (control) or with dapagliflozin (5 mg/kg body weight per day) for 8 or 25 weeks, respectively

Starting at 8 weeks of age, male homozygous *Ldlr*^*−/−*^ mice on a C57BL/6J-background (The Jackson Laboratory, Bar Harbor, ME, USA) received a high-fat, high-sucrose diabetogenic diet (DD, ssniff Spezialdiäten, Germany) supplemented without (control) or with 5 mg/kg body weight per day dapagliflozin (Forxiga 10 mg, Bristol-Myers Squibb/AstraZeneca EEIG, UK) for either 8 or 25 weeks (Fig. 1). Mice were allowed to drink and eat ad libitum and were kept on a normal 12 h light and dark cycle. This study was performed in accordance with the experimental animal use guidelines from the Deutsches Tierschutzgesetz and the Guide for the Care and Use of Laboratory Animals of the National Institutes of Health (Bethesda, MD, USA). All animal experiments were approved by the local research board for animal experimentation (LANUV; State Agency for Nature, Environment and Consumer Protection).

Electronic supplementary material (ESM) Table 1 shows a summary of vital parameters of mice after 25 weeks of treatment with dapagliflozin.

#### Randomisation and blinding—mice

Mice were randomised to different treatment groups. Investigators were blinded to group allocation for the analyses.

#### Metabolic measurements

The body weight of the mice was assessed weekly. Naso-anal length and body weight after 25 weeks of treatment were calculated to evaluate BMI. The body composition of lean and fat mass was assessed by NMR spectroscopy using the Minispec-mq7.5 (Bruker Corporation, Billerica, MA, USA). An oral glucose tolerance test was performed after 8 weeks of treatment. Mice were fasted for 6 h and fasting blood glucose values were assessed. Then animals received a 40% glucose solution to a final dose of 1 mg glucose/g body weight. Blood glucose values were measured after 0, 5, 15, 30, 60 and 120 min after glucose application using an ACCU-CHEK glucometer (Roche Diabetes Care Deutschland, Germany).

Blood pressure and heart rate were measured using a non-invasive, photoplethysmographic method (BP-2000 Blood Pressure Analysis System, version 2015.1.29, Visitech Systems, Apex, USA). The measurements were performed for 10 days in a row at the same time of day. There were three single measurements each day. For statistical analysis with the manufacturer’s software (BP-2000 Analysis Software) the mean values of the previous 3 days were used since the mice needed to become accustomed to the method in order to generate valid values. The systolic blood pressure was measured by a non-invasive computerised tail cuff system (Visitech System BP-2000, Apex, USA) as described previously [20].

#### Murine platelet activation

For flow cytometric analysis of platelet activation markers CD62P (P-selectin) and CD41/61 (activated glycoprotein IIb/IIIa), blood was drawn from *Ldlr*^*−/−*^ mice, collected in EDTA-coated tubes, washed twice and diluted 1:10 in tyrodes-buffer [21]. Afterwards, blood suspension was incubated with FITC-conjugated CD62P (clone Wug.E9) or phycoerythrin (PE)-conjugated CD41/61 (clone JON/A) monoclonal antibody (Emfret Analytics, Germany). Collagen-related peptide (CRP; 5 μg/ml, Dept of Biochemistry, University of Cambridge, Cambridge, UK) was used for platelet activation. The ex vivo effects of dapagliflozin in murine and human platelet-poor plasma (PPP) were tested after preincubation with dapagliflozin (0.5 μmol/l, Sigma-Aldrich Chemie, Germany) for 30 min at 37°C with or without HDL-cholesterol (5.2 mmol/l, Sigma-Aldrich) using PPP LOW-reagent, Thrombin Calibrator and FluCa-Kit (Diagnostics Stago, Asnières sur Seine, France). DMSO was used as an appropriate control. The samples were measured on a Gallios flow cytometer (Beckman Coulter, Germany). The data were analysed using Kaluza (version 2.1, 2018) flow analysis software (Beckman Coulter).

Electronic supplementary material (ESM) Table 2 shows the platelet characteristics.

#### Immune cell–platelet aggregate formation

For flow cytometry, murine blood was diluted in Tyrode’s buffer and washed twice via centrifugation at 650 *g* for 5 min. Afterwards, the centrifuged supernatant was removed and only the cell pellet was used for further analysis. The blood samples were incubated with antibodies for leucocytes (adenomatous polyposis coli protein [APC] anti-mouse CD45; BD-Bioscience), neutrophils (APC anti-mouse Ly6G, Biolegend [San Diego, CA, USA]) and platelets (PE anti-mouse glycoprotein [GP]Ib, EMFRET Analytics) for 15 min at room temperature. The reaction was stopped by adding PBS and the samples were analysed immediately on a FACSCalibur flow cytometer (BD Biosciences, San Jose, CA, USA). Only the mean fluorescence intensity of double-positive cells, indicating platelet/leucocyte or platelet–neutrophil aggregates, was evaluated.

#### Flow chamber experiments

Flow chamber experiments were performed as described previously [22]. Briefly, all blood samples were labelled with DylightX488 (EMFRET Analytics, #X488, 0.1 μg/ml) and perfused through the flow chamber on collagen-coated cover slips (200 μg/ml) at a shear rate of 1000/s. Platelet adhesion and thrombus formation were evaluated.

#### ATP release

For platelet preparation, murine heparin-anticoagulated blood was collected from the retro-orbital plexus and centrifuged at 304 *g* for 5 min at room temperature. To obtain platelet-rich plasma (PRP), the supernatant was centrifuged at 250 *g* for 6 min. PRP was washed at 650 *g* for 5 min at room temperature, and the resulting pellet was resuspended in Tyrode’s buffer supplemented with prostacyclin (0.5 μmol/l) and apyrase (0.02 U/ml). Before use, the platelets were resuspended in the same buffer supplemented with CaCl_2_(0.002 mol/l) and incubated at 37°C.

To determine ATP release, platelet suspension with 400,000 platelets/μl was incubated with CRP (5 μg/ml) for 2 min and fixed by adding formaldehyde (0.1%) and EDTA (3 mmol/l), then incubated for 1.5 h at room temperature and centrifuged at 14,196 *g* for 1 min. The supernatant was transferred into the same amount of 100% C_2_H_5_OH. The luminometric ATP release measurement was taken according to the ATP bioluminescence ELISA kit (Roche) protocol.

#### Tail bleeding

The mice were anaesthetised and the tail tip was cut with a scalpel at a defined diameter. The bleeding time was assessed by tracking the time from incision to bleeding-cessation [19].

#### Calibrated automated thrombography

Calibrated automated thrombography was used to determine endogenous thrombin generation in murine PPP according to the method described by Tchaikovski et al with slight modifications [19, 23].

The ex vivo effects of dapagliflozin in murine and human PPP were tested after preincubation with dapagliflozin (0.5 μmol/l, Sigma-Aldrich) for 30 min at 37°C with HDL-cholesterol (5.2 mmol/l, Sigma-Aldrich) using PPP LOW-reagent, Thrombin Calibrator and FluCa-Kit (Diagnostics Stago). The respective buffer without HDL was used as control sample.

Electronic supplementary material (ESM) Table 2 shows the platelet characteristics.

#### Plasma analysis

Cardiac blood was anticoagulated with a final concentration of 1 mmol/l EDTA followed by centrifugation for 15 min at 800 *g* and 4°C. The supernatant was centrifuged at 15,400 *g* and 4°C. Plasma concentrations of total circulating cholesterol, LDL-/VLDL-cholesterol and HDL-cholesterol were quantified using ELISA (HDL and LDL/VLDL Quantification Colorimetric/Fluorometric Kit, BioVision, Milpitas, CA, USA) according to the manufacturer’s protocol.

#### Plasma concentrations of cytokines and chemokines

Levels of inflammatory cytokines and chemokines were analysed using a commercially available multiplex-bead-based immunoassay (Bio-Plex Pro Mouse Cytokine 23-plex Assay, Biorad, Hercules, CA, USA). The analysis was performed using a Bio-Plex200 suspension array system (Biorad) according to the manufacturer’s instructions. Protein concentrations were calculated from the appropriate optimised standard curves with the help of the Bio-Plex-Manager-Software version 6.0 (Biorad). The samples were excluded if the concentration was below the range of measurement.

#### Measuring cholesterol from mouse faeces and bile

Mouse faeces were collected for 3 days. Bile was carefully removed from the gall bladder. Isolated lipids from mouse faeces were resuspended in 500 μl isopropanol/NP40(9:1), incubated at 37°C for 40 min and subsequently vigorously vortexed and centrifuged at room temperature (5 min, 376 *g*). The supernatant and undiluted bile were used for cholesterol measurement as described in the manufacturer’s protocol (Fluitest CHOL, Analyticon, Germany).

#### Quantitative real-time PCR

To analyse gene expression, livers and aortae were excised under RNase-free conditions from 33-week-old *Ldlr*^*−/−*^ mice and immediately stored at −80°C. Gene expression was assessed using real-time quantitative PCR (qPCR). 1000 ng total RNA was used for cDNA synthesis using QuantiTect reverse transcription kit (Qiagen, Germany) for RT-PCR. Gene expression was determined using real-time qPCR 7300 real-time PCR system (Thermo Fisher Scientific) and Platinum SYBR Green qPCR Super Mix UDG (Thermo Fisher Scientific, Waltham, MA, USA). Relative mRNA expression levels were compared using the ${2}^{-\Delta \Delta {\mathrm{C}}_{\mathrm{t}}}$ method. Primer sequences are given in Electronic supplementary material (ESM) Table 3.

#### Determining aortic plaque burden

To detect aortic lipid depositions, fixed aortas were stained with Oil Red O (Sigma-Aldrich). Atherosclerotic plaque burden in relation to the total aortic area was calculated using ImageJ software (ImageJ1.37v software, NIH, Bethesda, MD, USA).

#### Immunohistochemistry

For (immuno)histochemical analysis, 5 μm paraffin sections of the aortic root from fixed hearts were stained with H&E to calculate aortic root plaque size using three consecutive sections. Smooth muscle cell content within the atherosclerotic lesions was defined using a primary antibody against α-smooth muscle actin (1:300, Abcam, Cambridge, UK) and a horseradish peroxidase (HRP)-conjugated secondary antibody (1:400, goat-anti-rabbit IgG HRP, SantaCruz Biotechnologies, Dallas, TX, USA). For the analysis of plaque composition, hyaluronan (HA) was visualised by affinity histochemistry using biotinylated HA-binding-protein (Calbiochem, San Diego, CA, USA) and HRP-streptavidin conjugate (s5512, Sigma-Aldrich) for detection. In order to determine proteoglycan biglycan, the sections were pretreated with chondroitinase (chondroitinase ABC from Proteus vulgaris, Sigma-Aldrich) for 1 h at 37°C to expose epitopes of the core proteins. The biglycan core protein was stained with polyclonal antiserum against murine biglycan (1:500, rabbit, LF159) kindly provided by L. Fisher (National Institute of Dental and Craniofacial Research, NIH, Bethesda, MD, USA). Plaque infiltration by macrophages was determined using an antibody against Galectin-3 (Mac2; 1:1000, Cedarlane, Burlington, Canada) and an HRP-conjugated secondary antibody (1:1500, Goat-anti-rat IgG2a-HRP, NB7126, Novus Biological, Littleton, CO, USA). The detection procedure was followed by adding 3,3′-diaminobenzidine (DAB; Zytomed, Germany) as chromogen.

Pictures were taken with AxioImager.M2, AxioCam and AxioVS40V-4.8.2.0 software (Zeiss, Germany), and ImageJ software was used to quantify positively stained areas.

Electronic supplementary material (ESM) Table 4 shows plaque composition at the aortic root after 25 weeks of dapaflozin treatment.

#### Flow cytometric analysis of aortic immune cell composition

For flow cytometric analysis of aortic immune cells, the mice were euthanised after 25 weeks of treatment. Blood was collected from the heart and anticoagulated with 1 mmol/l EDTA. The analysis of the aorta was performed according to the protocol detailed by Butcher et al with slight modifications [19, 24]. Single cells were stained with the LIVE/DEAD Fixable Aqua Dead cell stain kit (Invitrogen Life Technologies, CA, USA) to exclude dead cells. To prevent unspecific binding, the cells were incubated with anti-CD16/32-antibody (clone93) before antibody staining with the following antibodies for detecting macrophages: F4/80-AF488 (cloneBM8), CD45-PE (clone30-F11), CD11b-PacBl (cloneM1/70). Unless otherwise indicated, antibodies were purchased from Biolegend. Absolute cell concentrations were determined using Flow-Count Fluorospheres (Beckman Coulter) according to the manufacturer’s instructions. In the case of lymphocyte analysis, erythrocytes were lysed with hypotonic ammonium chloride solution followed by centrifugation at 300 *g* for 10 min and 4°C. The cells were then resuspended in PBS containing 2 mmol/l EDTA and 0.5% BSA protein extraction buffer for staining. For the detection of neutrophils and monocytes including subsets, the blood samples were stained before lysis of erythrocytes using antibodies against the following surface molecules: Ly6C-AF488 (cloneHK1.4; Biolegend), CD11b-PE (cloneM1/70; BD Biosciences), CD115-APC (cloneAFS98; eBioscience, San Diego, CA, USA) and Ly6G-PacBl (clone1A8; Biolegend). Lymphocytes were separated with antibodies against the following surface molecules: CD4-FITC (cloneRM4-5, Invitrogen), CD45-PE (clone30-F11, Biolegend), CD8a-AF647 (clone53-6.7, Biolegend), CD3-APC/Cy7 (clone17A2, Biolegend), CD19-PacBl (clone6D5, Biolegend). To prevent unspecific binding, the cells were incubated with anti-CD16/32-antibody (clone93; Biolegend) before antibody staining. The samples were measured on Gallios flow cytometer (Beckman Coulter). The data were analysed using Kaluza flow analysis software (Beckman Coulter).

Murine blood cells counts after 8 and 25 weeks of dapagliflozin are given in Electronic supplementary material (ESM) Tables 5 and 6.

### Experiments including humans

#### Human blood sampling

Eight healthy volunteers received a daily oral dose of 10 mg dapagliflozin. The study was approved by the ethics committee of the Medical Faculty, Heinrich-Heine University Duesseldorf, Germany (Study-number: 5789R; registration ID 201612286).

This study complies with the Declaration of Helsinki, all participants gave informed consent. Electronic supplementary material (ESM) Table 7 shows the participants’ demographic characteristics. After 4 weeks of treatment, the participants’ blood was collected for light transmission aggregometry (LTA) and flow cytometry.

#### Randomisation and blinding—human

No randomisation was performed. Blinding of the investigators was carried out during performance of the experiments.

#### Human platelet CD62P-expression

Each blood sample was centrifuged at 250 *g* for 10 min to generate PRP. PRP was incubated with anti-CD41-FITC (IM0649U) and anti-CD62P-PE (IM1759U; both Beckman Coulter) antibodies. CRP (Dept. of Biochemistry, University of Cambridge, UK; 10 μg/ml) or TRAP-6 (Sigma-Aldrich; 10 μmol/l) were incubated for 30 min at 37°C. After fixation (4% formaldehyde, 10 min) the samples were measured on a Gallios flow cytometer. Kaluza flow analysis software was used for data analysis (both Beckman Coulter).

#### LTA

LTA was performed on PRP using a 2-channel aggregometer (LABiTec, Germany). ADP (5 μmol/l), TRAP-6 (10 μmol/l) (both Sigma-Aldrich) and collagen (Collagen Reagens HORM, NYCOMED, Linz, Austria; 10 μg/ml) were used to induce platelet aggregation followed by optical detection in a light channel as previously described [25]. Results are expressed as maximum of aggregation.

#### Determination of D-dimers

The blood samples were centrifuged at 2000 *g* for 15 min, kept at room temperature and were analysed within 4 h. Particle-enhanced immunoturbidimetric testing was conducted with Innovance D-Dimer assay (Siemens Healthcare Diagnostics, Germany) according to the manufacturer’s instructions.

### Statistical analysis

Statistical analyses were performed with GraphPad Prism 7 software (GraphPad Software, La Jolla, CA, USA). The data are presented as mean ± SD. Significance was evaluated by paired or unpaired two-tailed *t* test and one- or two-way ANOVA, with Tukey’s multiple comparison test. The respective statistical testing is stated in each figure legend and statistical significance was considered at *p* < 0.05.

## Results

### Reduced atherosclerosis in dapagliflozin-treated *Ldlr*^*−/−*^ mice

In order to study platelet function as a possible mechanism mediating dapagliflozin’s atheroprotective effects, we used a murine model of accelerated atherosclerosis and diet-induced insulin resistance. *Ldlr*^*−/−*^ mice received a DD diet either with or without 5 mg/kg body weight per day dapagliflozin. Two time points were chosen in order to detect early (8 weeks of feeding) and late (25 weeks of feeding) effects of dapagliflozin treatment (Fig. 1). Initial body weight, body weight gain, total body-fat and BMI did not differ between the two treatment groups (ESM Fig. 1a–c). Dapagliflozin treatment did not alter heart rate or blood pressure (ESM Fig. 1d–f). While fasting blood glucose was not affected (ESM Fig. 1g), dapagliflozin moderately improved glucose tolerance (ESM Fig. 1h, i). Accordingly, plasma insulin levels decreased (1663 ± 171.0 pmol/l vs 1274 ± 238.2 pmol/l, *p* < 0.05, ESM Fig. 1j).

**Fig. 2:**
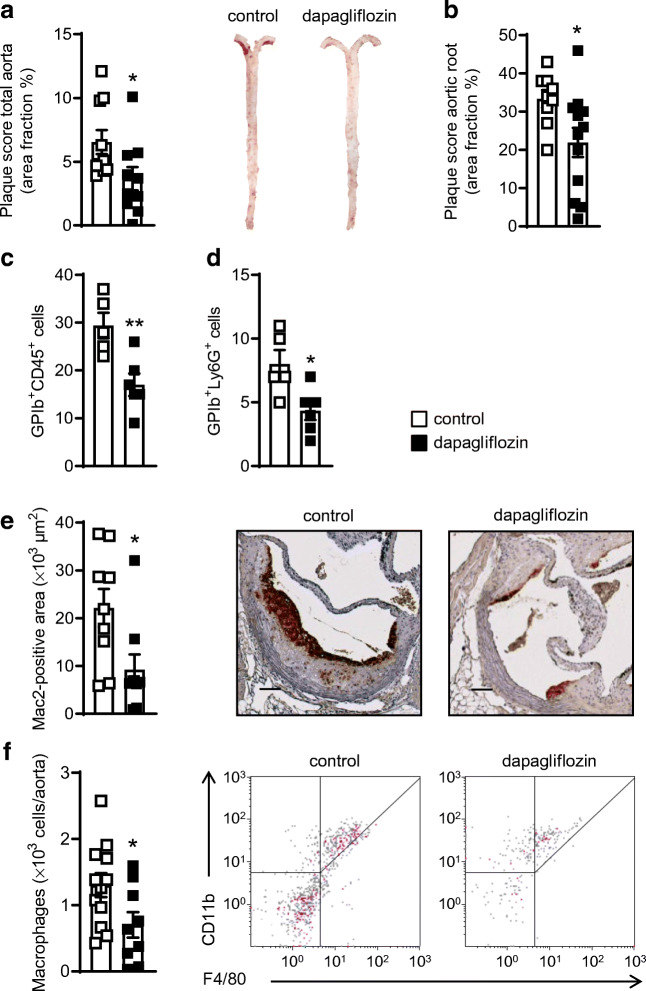
Dapagliflozin reduces atherosclerotic plaque burden, circulating platelet–leucocyte aggregates and macrophage infiltration in *Ldlr*^*−/−*^ mice. Male *Ldlr*^*−/−*^ mice received DD supplemented either without (control) or with dapagliflozin (5 mg/kg body weight per day) for 25 weeks followed by analysis of the atherosclerotic plaque burden and aortic immune cell infiltration. (**a**) Quantification of aortic plaque score (*n*=10) and representative images of Oil Red O-stained aortas. (**b**) Aortic root plaque size (*n*=9 control, *n*=12 dapagliflozin). (**c**) Platelet–leucocyte aggregates and (**d**) platelet–neutrophil aggregates in mice treated with dapagliflozin for 25 weeks or control mice (*n*=5 control, *n*=6 dapagliflozin). (**e**) Quantification of positive stained area for Mac2 (*n*=10) and representative images of immunohistochemical staining for Mac2. (**f**) Flow cytometric analysis of macrophages (CD45^+^CD11b^+^F4/80^+^) in the aortic wall (*n*=12 control, *n*=9 dapagliflozin) and representative flow cytometric dot plots. Data are presented as mean ± SD; unpaired Student’s *t* test: *p<0.05, **p<0.01 vs control. Scale bars represent 100 μm

Dapagliflozin-treated *Ldlr*^*−/−*^ mice had smaller aortic atherosclerotic lesions than controls after 25 weeks of treatment (Fig. 2a) and plaques at the aortic root were smaller (Fig. 2b). However, we observed no major changes in circulating cytokines/chemokines, neither after eight (ESM Fig. 2a) or 25 weeks of treatment (ESM Fig. 2b). Additionally, dapagliflozin did not affect the numbers of circulating leucocytes, lymphocytes, neutrophils and monocytes in mice analysed by flow cytometry (ESM Fig. 3). Representative gating schemes are shown in ESM Fig. 4*.*

**Fig. 3:**
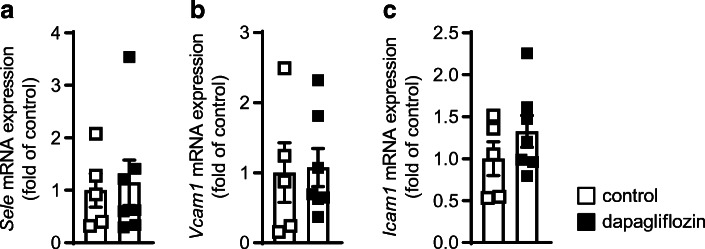
Dapagliflozin has no influence on aortic gene expression of leucocyte adhesion molecules in *Ldlr*^*−/−*^ mice. Male *Ldlr*^*−/−*^ mice received DD supplemented either without (control) or with dapagliflozin (5 mg/kg body weight per day) for 25 weeks. mRNA expression of (**a**) E-selectin (*Sele*)*,* (**b**) vascular cell adhesion molecule 1 (*Vcam1*) and (**c**) intercellular adhesion molecule 1 (*Icam1*) in the aorta of the respective treatment groups; *n*=5 control, *n*=7 dapagliflozin. Data are presented as mean ± SD; unpaired Student’s *t* test

Platelets have been described to exert important pro-atherogenic functions specifically by interacting with immune cells. Analysis of these platelet–leucocyte aggregates revealed that administering dapagliflozin decreased platelet–leucocyte (GPIb^+^CD45^+^: 29.40 ± 5.94 vs 17.00 ± 5.69 cells, *p* < 0.01; Fig. 2c) and platelet–neutrophil (GPIb^+^Ly6G^+^: 8.00 ± 2.45 vs 4.33 ± 1.75 cells, *p* < 0.05; Fig. 2d) aggregates, as detected by flow cytometry, thereby supporting the idea of decreased platelet-mediated leucocyte activation and recruitment. Accordingly, fewer lesional macrophages were detected by both immunohistochemical staining (Fig. 2e) and flow cytometric analysis of the aortic wall (1.31 ± 0.62 vs 0.70 ± 0.58 × 10^3^ cells/aorta, *p* < 0.01; Fig. 2f). A representative gating scheme for aortic macrophages is shown in ESM Fig. 5. We also analysed aortic gene expression of leucocyte adhesion molecules such as *Sele*, *Vcam1* and *Icam1* and found no alterations after treatment with dapagliflozin (Fig. 3a–c), pointing towards major effects on immune cells than on the aortic wall.

Further analysis of plaque composition revealed lower expression of biglycan (ESM Fig. 6a), smooth muscle cell actin (ESM Fig. 6b) and HA (ESM Fig. 6c) in dapagliflozin-treated animals compared with controls, pointing towards major changes in plaque composition, possibly based upon decreased lesion development.

### Dapagliflozin reduces alpha granule secretion in *Ldlr*^*−/−*^ mice

**Fig. 4:**
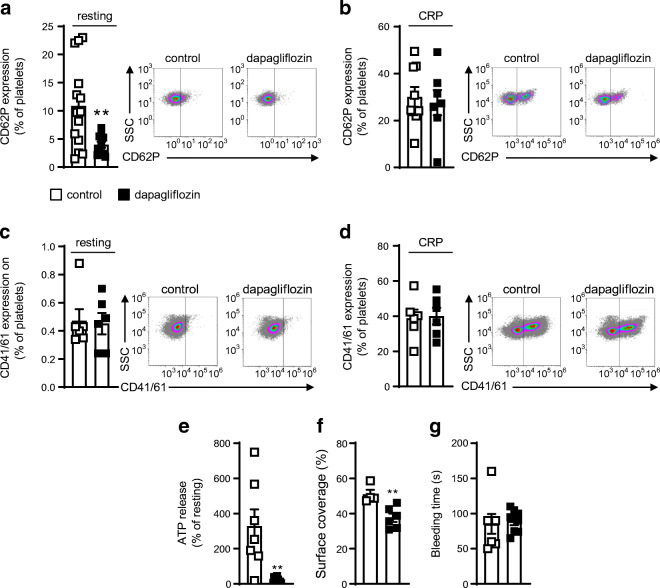
Dapagliflozin decreases murine platelet CD62P expression and platelet adhesion ex vivo. Male *Ldlr*^*−/−*^ mice received DD supplemented either without (control) or with dapagliflozin (5 mg/kg body weight per day) for 25 weeks. Quantification of CD62P expression on (**a**) resting platelets with representative dot plots of flow cytometric analyses (*n*=14 control, *n*=11 dapagliflozin) and (**b**) on murine platelets after stimulation with CRP (5 μg/ml) (*n*=9 control, *n*=7 dapagliflozin). Flow cytometric analysis of CD41/61 (activated glycoprotein IIb/IIIa) on (**c**) resting and (**d**) CRP-stimulated platelets (*n*=6). (**e**) Determination of ATP release after stimulation with CRP (5 μg/ml) was used to measure dense granule secretion in platelets isolated from mice of both treatment groups (*n*=7 control, *n*=10 dapagliflozin). (**f**) Flow chamber measurement was used to analyse thrombus formation ex vivo. Quantification of thrombus formation by determining surface coverage in flow chamber experiment (*n*=4 control, *n*=6 dapagliflozin). (**g**) Bleeding time after 25 weeks of treatment (*n*=8). Data are presented as mean ± SD; unpaired Student’s *t* test: **p<0.01 vs control

On the basis of these findings of altered platelet–leucocyte interaction, we focused in detail on platelet activation in dapagliflozin-treated mice. Neither absolute platelet counts and haematocrit (ESM Fig. 7a and b) nor the mean platelet volume (MPV; see ESM Table 4) were changed between treatment groups after 8 or 25 weeks of feeding. However, dapagliflozin decreased CD62P surface expression, thereby indicating less alpha granule secretion in resting platelets (*p* < 0.01, Fig. 4a). Stimulation by CRP revealed no differences between the two treatment groups (Fig. 4b). Unlike CD62P, CD41/CD61 (activated glycoprotein IIb/IIIa) expression after dapagliflozin treatment was not affected in either resting or stimulated platelets (Fig. 4c,d). In line with decreased release of alpha granules, dense granule secretion was reduced after administration of dapagliflozin as shown by measuring ATP release (*p* < 0.01, Fig. 4e). Likewise, less thrombus formation could be observed in the dapagliflozin-treated mice in a flow chamber experiment (*p* < 0.01, Fig. 4f). Despite reduced thrombus formation under flow conditions, however, bleeding time remained unchanged (85.29 ± 37.27 vs 89.25 ± 16.26 s, *p* = 0.78; Fig. 4g).

### Dapagliflozin reduces platelet CD62P expression ex vivo

In order to assess dapagliflozin’s direct effects, we analysed mouse blood samples from diabetic mice ex vivo before (control) or after incubation with dapagliflozin (0.5 μmol/l), resulting in a significant decrease in platelet CD62P expression (3.78 ± 1.20% vs 2.83 ± 1.06%, *p* < 0.01, ESM Fig. 8). This observation indicates that the compound has at least partial direct effects. However, isolated platelets showed no *Slc5a2* mRNA expression (data not shown), which suggests that the compound has possible off-target effects.

### Reduced thrombin generation in dapagliflozin-treated *Ldlr*^*−/−*^ mice

**Fig. 5:**
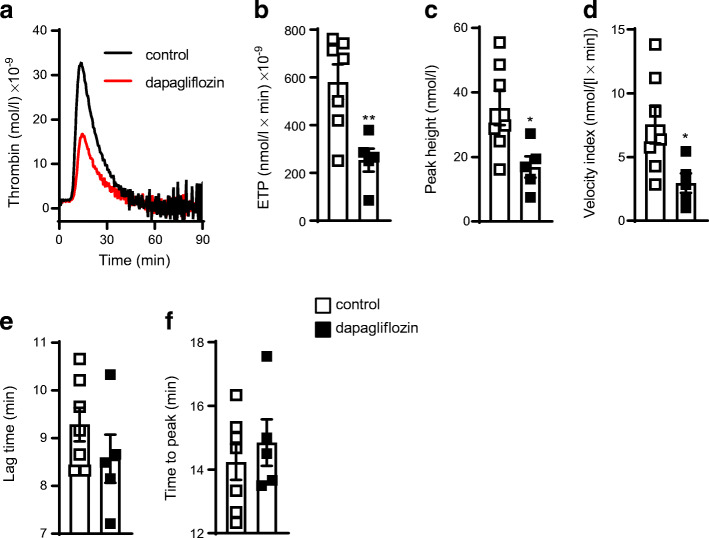
Dapagliflozin decreases thrombin generation in *Ldlr*^*−/−*^ mice. Male *Ldlr*^*−/−*^ mice received DD supplemented either without (control) or with dapagliflozin (5 mg/kg body weight per day) for 25 weeks. (**a**) Time course of endogenous thrombin generation and (**b**) calculation of the ETP as the respective AUC. Further kinetic parameters: (**c**) peak height; (**d**) velocity index; (**e**) lag time and (**f**) time to peak; *n*=7 control, *n*=5 dapagliflozin. Data are presented as mean ± SD; unpaired Student’s *t* test: *p<0.05 **p<0.01 vs control

Next, we investigated how dapagliflozin decreases alpha granule secretion in platelets. Thrombin is a major platelet-activator and an important driving force behind multiple inflammatory processes. Importantly, *Ldlr*^*−/−*^ mice treated with dapagliflozin for 25 weeks showed decidedly less thrombin generation (581.4 ± 194.6 nmol/l × min) × 10^−9^ thrombin vs 254.1 ± 106.4 (nmol/l × min) × 10^−9^ thrombin, measured as endogenous thrombin potential [ETP]; Fig. 5a,b). Further kinetic parameters of thrombin generation such as peak height (35.14 ± 14.03 nmol/l vs 16.87 ± 7.35 nmol/l, *p* < 0.05) and velocity index (7.55 ± 3.90 nmol/(l × min) vs 2.95 ± 1.71 nmol/(l × min), *p* < 0.05) were notably reduced after dapagliflozin treatment (Fig. 5c,d), while neither lag time nor time to peak differed significantly between the two treatment groups (Fig. 5e,f). The compound’s direct effects on thrombin generation were excluded because incubating platelet-free murine plasma with dapagliflozin (0.5 μmol/l) ex vivo did not affect thrombin generation (ESM Fig. 9).

### HDL-cholesterol increases after dapagliflozin treatment

**Fig. 6:**
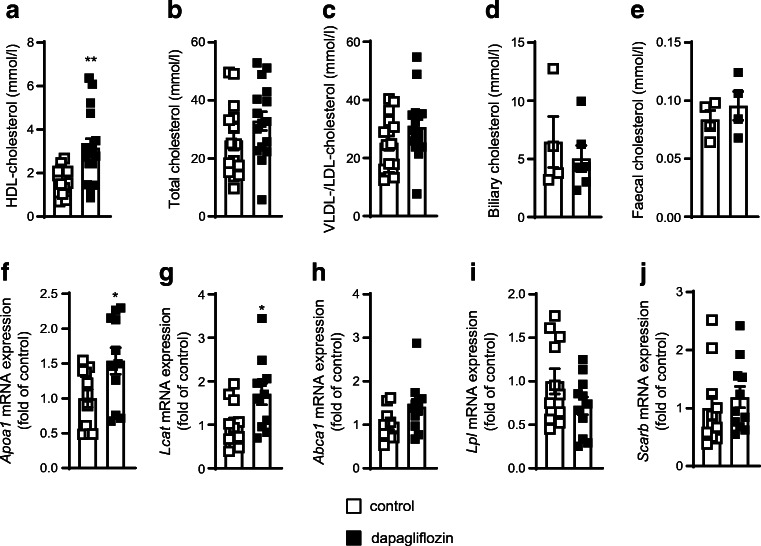
Treatment with dapagliflozin increases circulating HDL-cholesterol in *Ldlr*^*−/−*^ mice. Male *Ldlr*^*−/−*^ mice received DD supplemented either without (control) or with dapagliflozin (5 mg/kg body weight per day) for 25 weeks. Plasma analysis of (**a**) HDL-cholesterol, (**b**) total cholesterol and (**c**) VLDL-/LDL-cholesterol (*n*=15 control, *n*=16 dapagliflozin). (**d**) Biliary and (**e**) faecal cholesterol concentration (*n*=4 control, *n*=6 dapagliflozin). (**f**–**j**) Hepatic gene expression for HDL metabolism-associated genes after 25 weeks of dapagliflozin treatment (*n*=11). Data are presented as mean ± SD; (**a**–**e**) unpaired Student’s *t* test, (**f**–**j**) Mann–Whitney *U* test: *p<0.05 **p<0.01 vs control

To discover possible mechanisms for reduced thrombin generation, we analysed plasma cholesterol levels since HDL-cholesterol may interfere with thrombin generation by enhancing the anticoagulant activities of proteins C and S [16, 17]. In accordance with observations from clinical studies [26], dapagliflozin-treated mice showed distinctly higher plasma HDL-cholesterol (1.70 ± 0.58 mmol/l vs 3.15 ± 1.67 mmol/l, *p* < 0.01, Fig. 6a) while total and LDL/VLDL-cholesterol levels remained unaltered (Fig. 6b,c). Neither biliary nor faecal cholesterol excretion differed (Fig. 6d,e). However, we observed increased hepatic gene expression of *Apoa1* (which encodes apolipoprotein A1 [ApoA1]) and *Lcat* (encoding lecithin cholesterol acyltransferase [LCAT]), while no changes in the hepatic expression of *Scarb1* (encoding scavenger receptor class B member 1), *Lpl* (encoding lipoprotein lipase) and *Abca1* (encoding ATP-binding cassette transporter A1) could be detected (Fig. 6f–j).

### HDL-cholesterol decreases thrombin generation in human plasma

**Fig. 7:**
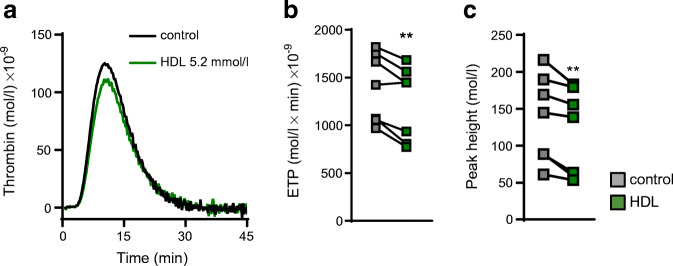
HDL directly decreases thrombin generation in human plasma. (**a**) Thrombin generation after incubating human PPP with 5.2 mmol/l HDL-cholesterol for 30 min at 37°C. (**b**) Quantification of ETP as the respective AUC and (**c**), peak height (*n*=7). Data are presented as paired values; paired *t* test: **p<0.01 vs control

Since dapagliflozin treatment increased HDL-cholesterol and decreased thrombin generation in mice, we tested for a potential causal relationship. Indeed, thrombin generation in human plasma clearly decreased after preincubation with human HDL-cholesterol (5.2 mmol/l): both the ETP and peak height were lower in the presence of HDL-cholesterol (Fig. 7a–c).

### Dapagliflozin inhibits alpha granule secretion in human platelets without affecting platelet aggregation

**Fig. 8:**
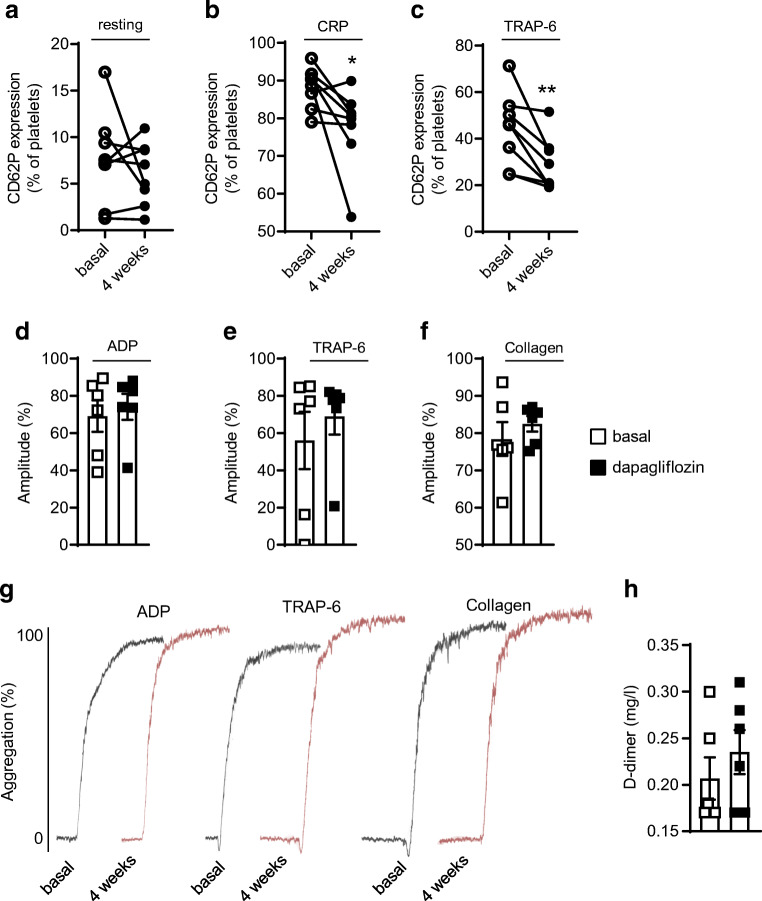
Dapagliflozin decreases CD62P expression on activated platelets in healthy volunteers. Eight healthy participants received a daily dose of 10 mg dapagliflozin orally for 4 weeks. Platelet CD62P expression was determined by flow cytometry before (basal) and after 4 weeks of treatment. CD62P expression on (**a**) resting platelets and on platelets after stimulation with (**b**) CRP (10 μg/ml) and (**c**) TRAP-6 (10 μmol/l) for 30 min at 37°C (*n*=8). LTA of platelets stimulated with (**d**) ADP (5 μmol/l), (**e**) TRAP-6 (10 μmol/l) and (**f**) collagen (10 μg/ml) (*n*=6). (**g**) Representative aggregation curves. (**h**) Blood concentration of D-dimer (*n*=6). Data are presented as paired values (**a**–**c**) or mean ± SD (**d**–**f**, **h**); paired *t* test: *p<0.05, **p<0.01

Finally, we aimed to see whether the underlying pathways affected by dapagliflozin in mice are also seen in humans treated with the SGLT2i. Healthy human volunteers received 10 mg dapagliflozin orally daily for 4 weeks and platelets were analysed by flow cytometry for CD62P expression as a marker of activated platelets. While resting platelets showed no changes (*p* = 0.38, Fig. 8a), dapagliflozin decreased CD62P expression after stimulation by CRP (88.13 ± 5.37% of platelets vs 77.59 ± 10.70%, *p* < 0.05) or TRAP-6 (44.23 ± 15.54% vs 28.96 ± 11.41%, *p* < 0.01; Fig. 8b,c) at baseline and after 4 weeks. Importantly, despite decreased alpha granule secretion, dapagliflozin did not affect platelet aggregation (Fig. 8d–f*)*. Representative aggregation curves are shown in Fig. 8g. Additionally, D-dimers were analysed to determine cross-linked fibrin degradation products in the blood after 4 weeks of treatment and revealed no effect of dapagliflozin (Fig. 8h).

**Fig. 9:**
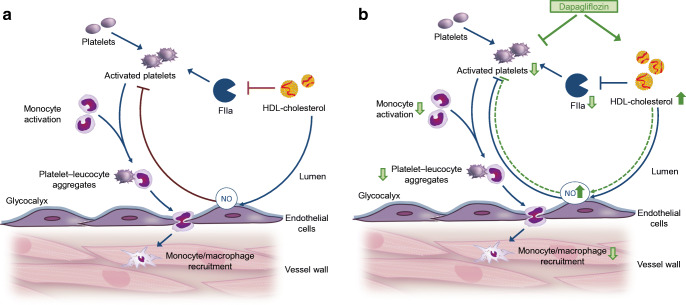
Proposed mechanism of dapagliflozin-mediated atheroprotection. Platelets are known drivers of atherosclerosis and atherothrombosis. Disturbed platelet activation has also been suggested to occur in type 2 diabetes. (**a**) Interplay between platelet function, thrombin generation and inflammation in atherogenesis. (**b**) Direct inhibitory effect of dapagliflozin on isolated platelets and increased HDL-cholesterol levels: dapagliflozin decreases thrombin-mediated platelet activation and alpha granule secretion via both direct effects and HDL-mediated effects on thrombin formation. Decreased platelet–leucocyte aggregates and subsequent diminished monocyte–macrophage-recruitment to the vascular wall contribute to atheroprotection by dapagliflozin treatment

In sum, the results of this study indicate that dapagliflozin decreases thrombin-mediated platelet activation and alpha granule secretion via both direct effects and HDL-mediated effects on thrombin formation. Thus, decreased platelet–leucocyte aggregates and subsequent diminished monocyte–macrophage-recruitment to the vascular wall contribute to atheroprotection by dapagliflozin treatment (Fig. 9).

## Discussion

In the present study we show that atheroprotective effects of dapagliflozin are mediated by: (1) increases in HDL-cholesterol, which in turn (2) reduce thrombin generation, (3) alleviate platelet alpha granule secretion and (4) decrease platelet–leucocyte aggregates without affecting bleeding time in mice. As a consequence, dapagliflozin lowers macrophage infiltration and atherosclerotic plaque burden. Our main finding—decreased surface expression of the platelet activation marker CD62P—could be in part translated to humans where dapagliflozin application decreased, after agonist-specific stimulation, the number of CD62P-positive platelets without changing platelet aggregation.

Cardiovascular disease in individuals with diabetes remains the principal cause of disability and death due to the manifestation of coronary artery heart disease, myocardial infarction, ischaemic stroke, peripheral artery disease and heart failure. There are multiple mechanisms for the increased risk of atherosclerotic cardiovascular disease, including hyperglycaemia, insulin resistance, dyslipidaemia, inflammation, generation of reactive oxygen species and endothelial dysfunction, promoting atherogenesis, vascular calcification and hypercoagulability [7].

In order to address the important impact of glucose-lowering treatment on cardiovascular risk in patients with type 2 diabetes, cardiovascular outcome trials not only evaluate the glucose-lowering effect but also possible cardiovascular benefits of diabetes medications.

Our data showing atheroprotective effects of dapagliflozin are in line with previous experimental and clinical studies using other SGLT2is. In *ApoE*-deficient mice dapagliflozin inhibited interleukin-1β secretion by macrophages and reduced atherosclerosis via the ROS-NLRP3-caspase-1 pathway [27]. In *Ldlr*-deficient mice, SGLT2is improved lipoprotein clearance exclusive of LDL receptor, thereby decreasing atherosclerotic plaque burden [28]. In humans, the EMPA-REG OUTCOME cardiovascular outcome trial demonstrated significantly decreased cardiovascular-caused deaths by empagliflozin [29]. In addition, dapagliflozin’s favourable impact on reversing hypertension and left-ventricular remodelling were confirmed in humans: the DECLARE–TIMI 58 trial showed a lower rate of cardiovascular deaths and fewer hospitalisations for heart failure due to decreased occurrence of reduced ejection fraction in patients treated with dapagliflozin compared with placebo [11].

However, despite these beneficial effects in experimental and clinical studies the underlying mechanism has not yet been defined. It has been suggested that SGLT2 inhibition may protect against cardiovascular diseases by factors that probably do not directly depend on curtailing blood glucose but may include reduced blood pressure, weight loss and increased HDL-cholesterol [29].

Our findings describe a novel mechanism for SGLT2is that might reduce cardiovascular risk in people with type 2 diabetes during long-term treatment, and we identify HDL-cholesterol and thrombin as crucial factors in this process. We provide evidence that the reduction of thrombin generation and platelet activation by dapagliflozin is a crucial factor in the discussion of possible mechanisms contributing to SGLT2is’ contribution to a beneficial cardiovascular risk profile. Here, the reduced platelet activation, in combination with decreased atherosclerosis, favourably influences plaque composition and blood lipid profile.

Of note, the dapagliflozin dose we have chosen did not at all affect body weight or blood pressure and only moderately affected blood glucose homeostasis. Beneficial effects of dapagliflozin treatment on hyperglycaemia were primarily observed by improved glucose tolerance and decreased insulin plasma levels, but fasting blood glucose was unchanged. Additionally, in contrast to previous reports, our dose had no effect on circulating immune cells or inflammatory cytokines [30]. Accordingly, in the present study, dapagliflozin decreased alpha granule secretion in healthy humans, in whom the compound does not affect blood glucose levels [31], therefore excluding this as the responsible mechanism.

In line with our findings, previous clinical trials have shown that dapagliflozin causes increases in HDL-cholesterol in the early and late treatment phases [30, 31]. Epidemiological studies clearly demonstrated a correlation between elevated plasma HDL-cholesterol levels and cardiovascular events [32]. However, interventional studies generated conflicting results, and the complex underlying mechanisms of HDL-mediated effects on atherogenesis and atheroprogression need further investigation. It has become clear that, instead of therapeutically trying to increase absolute HDL levels, first a detailed analysis and mechanical understanding of overall HDL composition and function is crucial. Regarding the possible mechanisms through which dapagliflozin mediates HDL-cholesterol increase, we detected increased hepatic gene expression of *Apoa1* and *Lcat* after treatment with dapagliflozin, both known to be involved in HDL metabolism and probably contributing to increased HDL cholesterol levels [33, 34]. Indeed, previous preclinical studies have shown that ApoA1, the major apolipoprotein of HDL, has atheroprotective functions [35], a result confirmed in first clinical trials using different ApoA1-based strategies [36]. By contrast, the role of LCAT, which is of major importance in HDL-maturation and plasma HDL levels, is controversial as regards its possible involvement in atherosclerosis [33, 37]. However, the contradictory results from multiple animal and human studies probably reflect HDL particles’ huge heterogeneity, which determines their functional outcome [38].

In addition, HDL shows further promising anti-atherogenic potential due to interaction with platelets, which are also involved in early and late stages of atherosclerosis. Here we provide evidence that HDL directly reduces thrombin generation, possibly by enhancing the anticoagulant protein C pathway [16, 39]. Increased HDL-cholesterol thereby additionally supports dapagliflozin-mediated inhibition of thrombin generation and platelet activation because HDL’s antithrombotic properties include attenuation of both thrombin generation and CD62P-expression [39, 40].

The data presented here show that dapagliflozin also directly influences CD62P expression independent of HDL and thrombin in a mouse model of diabetes and directly affects alpha- and dense granule secretion. However, these direct effects were not observed in healthy humans. Until now, extrarenal SGLT2 expression was reported only in the pancreas [41], not in platelets, and in our study, no SGLT2 expression could be detected in isolated platelets. Therefore, directly reducing CD62P expression on platelets ex vivo might be a novel off-target effect of dapagliflozin treatment.

Platelet activation (granule release, integrin activation) and thrombus formation requires Ca^2+^ mobilisation [42]; it can therefore be assumed that dapagliflozin reduces platelet granule release as well as the formation of platelet–leucocyte aggregates, probably due to a decrease in intracellular Ca^2+^ levels, as SGLT2is can inhibit Na^+^/H^+^ exchanger leading to reduced Ca^2+^ mobilisation [43].

It is well known that platelet function changes under diabetic conditions and the risk of thrombotic events is increased. While results from studies in diabetic volunteers are in part controversial [14, 15, 44], we show here that dapagliflozin affects resting platelets in diabetic mice. However, this is in contrast to healthy humans, in whom an effect was only detectable after agonist stimulation. It might be suggested that platelet pre-activation, as in obesity and insulin resistance, is needed to see dapagliflozin’s effects [45]. Clearly, translation to volunteers with diabetes is required in further studies, particularly against the background that clinical studies have shown controversial results regarding platelet activation under diabetic conditions [14, 15].

Activated platelets facilitate leucocyte adhesion to the endothelium and subsequent immune cell recruitment to the vascular wall [46]. It is notable that platelet–leucocyte aggregates have been identified as important factors driving atherothrombosis and atherosclerosis but also myocardial dysfunction and heart failure [47]. Therefore, the observed decrease in platelet–leucocyte aggregates by dapagliflozin further confirms the hypothesis of reduced atherogenesis due to less platelet-mediated inflammation and implicates clinical impact on cardiovascular events. However, further studies are needed to provide evidence that the pathway described here also translates to the pathophysiology in diabetic patients.

In summary, we report here that dapagliflozin treatment, directly and indirectly through HDL, decreases thrombin generation and platelet granule secretion without interfering with haemostasis. This newly identified mechanism reduces plaque inflammation and decelerates atherosclerotic progression even without affecting fasting blood glucose levels in mice and therefore can be interpreted as a blood glucose independent or pleiotropic effect of dapagliflozin. For diabetic individuals with increased risk of cardiovascular events, these findings might be of major clinical importance since other glucose-lowering compounds lack cardiovascular benefits in clinical trials.

## Supplementary Information

ESM (PDF 1.26 mb)

## Data Availability

Raw data generated and/or analysed during the current study are available from the corresponding author upon reasonable request.

## Abbreviations

APC: Adenomatous polyposis coli protein
ApoA1: Apolipoprotein A1
CRP: Collagen-related peptide
DD: Diabetogenic diet
DECLARE–TIMI 58: Dapagliflozin Effect on Cardiovascular Events–Thrombolysis in Myocardial Infarction 58
ETP: Endogenous thrombin potential
GP: Glycoprotein
HA: Hyaluronan
HRP: Horseradish peroxidase
LCAT: Lecithin cholesterol acyltransferase
LTA: Light transmission aggregometry
Ly6G: Lymphocyte antigen 6 complex, locus G
NLRP3: Nucleotide-binding oligomerisation domain, leucine-rich repeat, and pyrin domain-containing 3
PE: Phycoerythrin
PPP: Platelet-poor plasma
PRP: Platelet-rich plasma
qPCR: Quantitative PCR
SGLT2: Sodium–glucose cotransporter 2
SGLT2i: SGLT2 inhibitor
TRAP-6: Thrombin receptor activator peptide-6

## References

[1] Oku A, Ueta K, Arakawa K (1999). T-1095, an inhibitor of renal Na+-glucose cotransporters, may provide a novel approach to treating diabetes. Diabetes.

[2] Bailey CJ, Gross JL, Hennicken D, Iqbal N, Mansfield TA, List JF (2013). Dapagliflozin add-on to metformin in type 2 diabetes inadequately controlled with metformin: a randomized, double-blind, placebo-controlled 102-week trial. BMC Med.

[3] Henry RR, Murray AV, Marmolejo MH, Hennicken D, Ptaszynska A, List JF (2012). Dapagliflozin, metformin XR, or both: initial pharmacotherapy for type 2 diabetes, a randomised controlled trial. Int J Clin Pract.

[4] Chao EC (2014). SGLT-2 Inhibitors: A New Mechanism for Glycemic Control. Clin Diabetes.

[5] Dauriz M, Targher G, Laroche C (2017). Association Between Diabetes and 1-Year Adverse Clinical Outcomes in a Multinational Cohort of Ambulatory Patients With Chronic Heart Failure: Results From the ESC-HFA Heart Failure Long-Term Registry. Diabetes Care.

[6] Seferovic PM, Coats AJS, Ponikowski P (2020). European Society of Cardiology/Heart Failure Association position paper on the role and safety of new glucose-lowering drugs in patients with heart failure. Eur J Heart Fail.

[7] Low Wang CC, Hess CN, Hiatt WR, Goldfine AB (2016). Clinical Update: Cardiovascular Disease in Diabetes Mellitus: Atherosclerotic Cardiovascular Disease and Heart Failure in Type 2 Diabetes Mellitus - Mechanisms, Management, and Clinical Considerations. Circulation.

[8] Nabel EG, Braunwald E (2012). A tale of coronary artery disease and myocardial infarction. N Engl J Med.

[9] Action to Control Cardiovascular Risk in Diabetes Study Group, Gerstein HC, Miller ME, Byington RP et al (2008) Effects of intensive glucose lowering in type 2 diabetes. N Engl J Med 358(24):2545–2559. 10.1056/NEJMoa0802743 10.1056/NEJMoa0802743 PMC4551392 18539917

[10] UK Prospective Diabetes Study (UKPDS) Group (1999) Intensive blood-glucose control with sulphonylureas or insulin compared with conventional treatment and risk of complications in patients with type 2 diabetes (UKPDS 33). Lancet 352(9131): 837–853 9742976

[11] Wiviott SD, Raz I, Bonaca MP (2019). Dapagliflozin and Cardiovascular Outcomes in Type 2 Diabetes. N Engl J Med.

[12] Schreyer SA, Vick C, Lystig TC, Mystkowski P, LeBoeuf RC (2002). LDL receptor but not apolipoprotein E deficiency increases diet-induced obesity and diabetes in mice. Am J Physiol Endocrinol Metab.

[13] Neuhofer A, Wernly B, Leitner L (2014). An accelerated mouse model for atherosclerosis and adipose tissue inflammation. Cardiovasc Diabetol.

[14] Rodriguez BAT, Johnson AD (2020). Platelet Measurements and Type 2 Diabetes: Investigations in Two Population-Based Cohorts. Front Cardiovasc Med.

[15] Serebruany V, Pokov I, Kuliczkowski W, Chesebro J, Badimon J (2008). Baseline platelet activity and response after clopidogrel in 257 diabetics among 822 patients with coronary artery disease. Thromb Haemost.

[16] Lerch PG, Spycher MO, Doran JE (1998). Reconstituted high density lipoprotein (rHDL) modulates platelet activity in vitro and ex vivo. Thromb Haemost.

[17] Nofer JR, Walter M, Kehrel B (1998). HDL3-mediated inhibition of thrombin-induced platelet aggregation and fibrinogen binding occurs via decreased production of phosphoinositide-derived second messengers 1,2-diacylglycerol and inositol 1,4,5-tris-phosphate. Arterioscler Thromb Vasc Biol.

[18] Aoki I, Shimoyama K, Aoki N (1996). Platelet-dependent thrombin generation in patients with diabetes mellitus: effects of glycemic control on coagulability in diabetes. J Am Coll Cardiol.

[19] Grandoch M, Kohlmorgen C, Melchior-Becker A (2016). Loss of Biglycan Enhances Thrombin Generation in Apolipoprotein E-Deficient Mice: Implications for Inflammation and Atherosclerosis. Arterioscler Thromb Vasc Biol.

[20] Nagy N, Melchior-Becker A, Fischer JW (2010). Long-term treatment with the AT1-receptor antagonist telmisartan inhibits biglycan accumulation in murine atherosclerosis. Basic Res Cardiol.

[21] Donner L, Falker K, Gremer L (2016). Platelets contribute to amyloid-beta aggregation in cerebral vessels through integrin alphaIIbbeta3-induced outside-in signaling and clusterin release. Sci Signal.

[22] Gowert NS, Kruger I, Klier M (2017). Loss of Reelin protects mice against arterial thrombosis by impairing integrin activation and thrombus formation under high shear conditions. Cell Signal.

[23] Tchaikovski SN, Vanv BJ, Rosing J, Tans G (2007). Development of a calibrated automated thrombography based thrombin generation test in mouse plasma. J Thromb Haemost.

[24] Butcher MJ, Herre M, Ley K, Galkina E (2011). Flow cytometry analysis of immune cells within murine aortas. J Vis Exp.

[25] Born GV (1962). Aggregation of blood platelets by adenosine diphosphate and its reversal. Nature.

[26] Bailey CJ, Gross JL, Pieters A, Bastien A, List JF (2010). Effect of dapagliflozin in patients with type 2 diabetes who have inadequate glycaemic control with metformin: a randomised, double-blind, placebo-controlled trial. Lancet.

[27] Leng W, Ouyang X, Lei X (2016). The SGLT-2 Inhibitor Dapagliflozin Has a Therapeutic Effect on Atherosclerosis in Diabetic ApoE(-/-) Mice. Mediat Inflamm.

[28] Al-Sharea A, Murphy AJ, Huggins LA, Hu Y, Goldberg IJ, Nagareddy PR (2018). SGLT2 inhibition reduces atherosclerosis by enhancing lipoprotein clearance in Ldlr(-/-) type 1 diabetic mice. Atherosclerosis.

[29] Zinman B, Wanner C, Lachin JM (2015). Empagliflozin, Cardiovascular Outcomes, and Mortality in Type 2 Diabetes. N Engl J Med.

[30] Nagareddy PR, Murphy AJ, Stirzaker RA (2013). Hyperglycemia promotes myelopoiesis and impairs the resolution of atherosclerosis. Cell Metab.

[31] Komoroski B, Vachharajani N, Boulton D (2009). Dapagliflozin, a novel SGLT2 inhibitor, induces dose-dependent glucosuria in healthy subjects. Clin Pharmacol Ther.

[32] Gordon DJ, Probstfield JL, Garrison RJ (1989). High-density lipoprotein cholesterol and cardiovascular disease. Four prospective American studies. Circulation.

[33] Ossoli A, Pavanello C, Calabresi L (2016). High-Density Lipoprotein, Lecithin: Cholesterol Acyltransferase, and Atherosclerosis. Endocrinol Metab.

[34] Rosenson RS, Brewer HB, Chapman MJ (2011). HDL measures, particle heterogeneity, proposed nomenclature, and relation to atherosclerotic cardiovascular events. Clin Chem.

[35] Paszty C, Maeda N, Verstuyft J, Rubin EM (1994). Apolipoprotein AI transgene corrects apolipoprotein E deficiency-induced atherosclerosis in mice. J Clin Invest.

[36] Smith JD (2010). Apolipoprotein A-I and its mimetics for the treatment of atherosclerosis. Curr Opin Investig Drugs.

[37] Nakamura Y, Kotite L, Gan Y, Spencer TA, Fielding CJ, Fielding PE (2004). Molecular mechanism of reverse cholesterol transport: reaction of pre-beta-migrating high-density lipoprotein with plasma lecithin/cholesterol acyltransferase. Biochemistry.

[38] Calabresi L, Gomaraschi M, Franceschini G (2010). High-density lipoprotein quantity or quality for cardiovascular prevention?. Curr Pharm Des.

[39] Griffin JH, Kojima K, Banka CL, Curtiss LK, Fernandez JA (1999). High-density lipoprotein enhancement of anticoagulant activities of plasma protein S and activated protein C. J Clin Invest.

[40] Mineo C, Deguchi H, Griffin JH, Shaul PW (2006). Endothelial and antithrombotic actions of HDL. Circ Res.

[41] Bonner C, Kerr-Conte J, Gmyr V (2015). Inhibition of the glucose transporter SGLT2 with dapagliflozin in pancreatic alpha cells triggers glucagon secretion. Nat Med.

[42] Elvers M, Herrmann A, Seizer P (2012). Intracellular cyclophilin A is an important Ca(2+) regulator in platelets and critically involved in arterial thrombus formation. Blood.

[43] Uthman L, Baartscheer A, Bleijlevens B (2018). Class effects of SGLT2 inhibitors in mouse cardiomyocytes and hearts: inhibition of Na(+)/H(+) exchanger, lowering of cytosolic Na(+) and vasodilation. Diabetologia.

[44] Kring C, Rasmussen LM, Lindholt JS, Diederichsen ACP, Vinholt PJ (2020). Platelet aggregation is not altered among men with diabetes mellitus. Acta Diabetol.

[45] Eibl N, Krugluger W, Streit G, Schrattbauer K, Hopmeier P, Schernthaner G (2004). Improved metabolic control decreases platelet activation markers in patients with type-2 diabetes. Eur J Clin Investig.

[46] Huo Y, Schober A, Forlow SB (2003). Circulating activated platelets exacerbate atherosclerosis in mice deficient in apolipoprotein E. Nat Med.

[47] Glezeva N, Gilmer JF, Watson CJ, Ledwidge M (2016). A Central Role for Monocyte-Platelet Interactions in Heart Failure. J Cardiovasc Pharmacol Ther.

