# Supplementary material for: Dapagliflozin reduces thrombin generation and platelet activation: implications for cardiovascular risk reduction in type 2 diabetes mellitus
Source: Diabetologia. 2021 Jun 16;64(8):1834–49. doi: 10.1007/s00125-021-05498-0 (PMC8245397; doi:10.1007/s00125-021-05498-0)
Supplement: Supplementary file 1 — (PDF 1.26 mb) [file 125_2021_5498_MOESM1_ESM.pdf]

	Control	Dapagliflozin	*P value*
Body weight ($x10^{-3}$ kg)	49.9 ± 5.8	50.4 ± 6.4	0.839
Total body fat mass (fat mass / total mass)	0.36 ± 0.007	0.37 ± 0.014	0.607
BMI ($x10^{-2}$ g/mm^2)	0.53 ± 0.03	0.53 ± 0.04	0.852
Heart rate (bpm)	657 ± 21	666 ± 33	0.588
Systolic blood pressure (mmHg)	117 ± 6	116 ± 9	0.898
Diastolic blood pressure (mmHg)	58 ± 9	51 ± 9	0.279
Fasting blood glucose / 0 minutes (mmol/l)	15.1 ± 1.4	15.3 ± 1.3	0.808
Fasting blood glucose / 5 minutes (mmol/l)	21.9 ± 1.0	21.9 ± 1.6	0.953
Fasting blood glucose / 15 minutes (mmol/l)	27.7 ± 2.4	24.7 ± 1.9	0.040
Blood glucose, oGTT 30 minutes (mmol/l)	22.2 ± 3.4	20.7 ± 3.5	0.455
Blood glucose, oGTT 60 minutes (mmol/l)	18.0 ± 3.3	14.9 ± 1.3	0.056
Blood glucose, oGTT 90 minutes (mmol/l)	16.3 ± 1.4	14.7 ± 1.0	0.040
AUC (mmo/l * min)	2367 ± 215	2118 ± 210	0.147
Insulin (pmol/l)	1663 ± 171	1274 ± 238	0.038

ESM Table 1. Vital parameter of mice after 25 weeks of treatment
oGTT: oral glucose tolerance test

	Control	Dapagliflozin	*p - value*
Platelets ($10^3/mm^3$)	952 ± 258	971 ± 276	0.895
Hematocrit (%)	53 ± 5.4	54 ± 5.9	0.756
Mean platelet volume (MPV) 8 weeks	5.2 ± 0.1	5.3 ± 0.1	0.713
Mean platelet volume (MPV) 25 weeks	5.2 ± 0.2	5.2 ± 0.3	0.143
CD62P expression (% of platelets)	3.8 ± 1.2	2.8 ± 1.1	0.005
ETP AUC (nM*min)	486 ± 46	485 ± 50	0.802
Thrombin peak height (nM)	86.5 ± 7.3	87.4 ± 7.7	0.232

ESM Table 2. Murine platelet characterics and thrombin generation

Gene	Forward primer (5’-3’)	Reverse primer (5’-3’)
Abca1	*CTACCAACCTGCCCGTTCTA*	*ATGCCGATGAAGAGGTTCAC*
Apoa1	*TATGTGGATGCGGTCAAAGA*	*CTGCAGCTGACTAACGGTTG*
Apoa2	*GACGGACCGGATATGCAGAG*	*AGCTGCTCGTGTGTCTTCTC*
Icam1	*TTCTCATGCCGCACAGAACT*	*TCCTGGCCTCGGAGACATTA*
Lcat	*CTTCACCATCTGGCTGGATT*	*GCCCAGAGCTGTGGTTGTAG*
18S	*GCAATTATTCCCCATGAACG*	*GGCCTCACTAAACCATCCAA*
Lpl	*TCGTCATCGAGAGGATCCGA*	*ACACTGCTGAGTCCTTTCCC*
Scarb1	*CACCCTTCATGACACCCGAA*	*TGGCAAACAGAGTATCGGGG*
Sele	*CGAGACGCCATCATGCAAAG*	*CCTGCAACGTGAAACTCTGC*
Vcam1	*GTCACGGTCAAGTGTTTGGC*	*TGTTCATGAGCTGGTCACCC*

ESM Table 3. Primer sequences
Abca1: *ATP-binding cassette transporter A1; Apoa1: Apolipoprotein A1; Apoa2: Apolipoprotein A2; Icam1: Intercellular adhesion molecule 1; Lcat: Lecithin cholesterol acyltransferase; 18S:18S ribosomal RNA; Lpl: lipoprotein lipase; Scarb1: Scavenger Receptor Class B Member 1; Sele: selectin E; Vcam1: Vascular cell adhesion protein 1.*

	**Control**	**Dapagliflozin**	***p - value***
Biglycan (x10^3 um^2)	23.5 ± 11.0	10.3 ± 10.8	0.017
aSMA (x10^3 um^2)	7.7 ± 3.4	2.7 ± 2.9	0.003
Hyaluronan (x10^3 um^2)	43.0 ± 15.8	20.2 ± 17.2	0.008

ESM Table 4. Plaque composition at the aortic root after 25 weeks of treatment with dapagliflozin
aSMA: alpha-smooth muscle actin

	**Control**	**Dapagliflozin**	***p - value***
Leucocytes (x10^3 cells/ul blood)	4.5 ± 2.5	3.6 ± 1.5	0.287
Lymphocytes (x10^3 cells/ul blood)	3.0 ± 1.8	2.3 ± 0.8	0.253
Neutrophils (x10^3 cells/ul blood)	0.3 ± 0.3	0.2 ± 0.1	0.299
Monocytes (x10^3 cells/ul blood)	0.3 ± 0.3	0.3 ± 0.1	0.447
Ly6C^{high} monocytes (cells/ul blood)	112 ± 131	77 ± 52	0.382
Ly6C^{low} monocytes (cells/ul blood)	138 ± 92	126 ± 69	0.715

ESM Table 5. Murine blood cell count after 8 weeks of treatment with dapagliflozin

	**Control**	**Dapagliflozin**	***p - value***
Leucocytes (x10^3 cells/ul blood)	5.3 ± 2.8	6.2 ± 4.9	0.563
Lymphocytes (x10^3 cells/ul blood)	4.1 ± 1.8	5.1 ± 2.7	0.402
Neutrophils (x10^3 cells/ul blood)	0.8 ± 0.8	0.6 ± 0.5	0.596
Monocytes (x10^3 cells/ul blood)	1.0 ± 1.2	1.0 ± 1.1	0.937
Ly6C^{high} monocytes (cells/ul blood)	41 ± 46	49 ± 47	0.719
Ly6C^{low} monocytes (cells/ul blood)	33 ± 38	46 ± 41	0.482

ESM Table 6. Murine blood cell count after 25 weeks of treatment with dapagliflozin

	**Sex**<br><br>**(m = male, f = female)**	**Age**<br><br>**(years)**	**Mean age**<br><br>**(**male versus female = n.s.)
Healthy volunteer 1	m	42	**34** ± **9**
Healthy volunteer 2	m	51	
Healthy volunteer 3	m	29	
Healthy volunteer 4	m	28	
Healthy volunteer 5	f	28	**38** ± **11**
Healthy volunteer 6	f	29	
Healthy volunteer 7	f	39	
Healthy volunteer 8	f	27	

ESM Table 7. Demographic characteristics of healthy volunteers

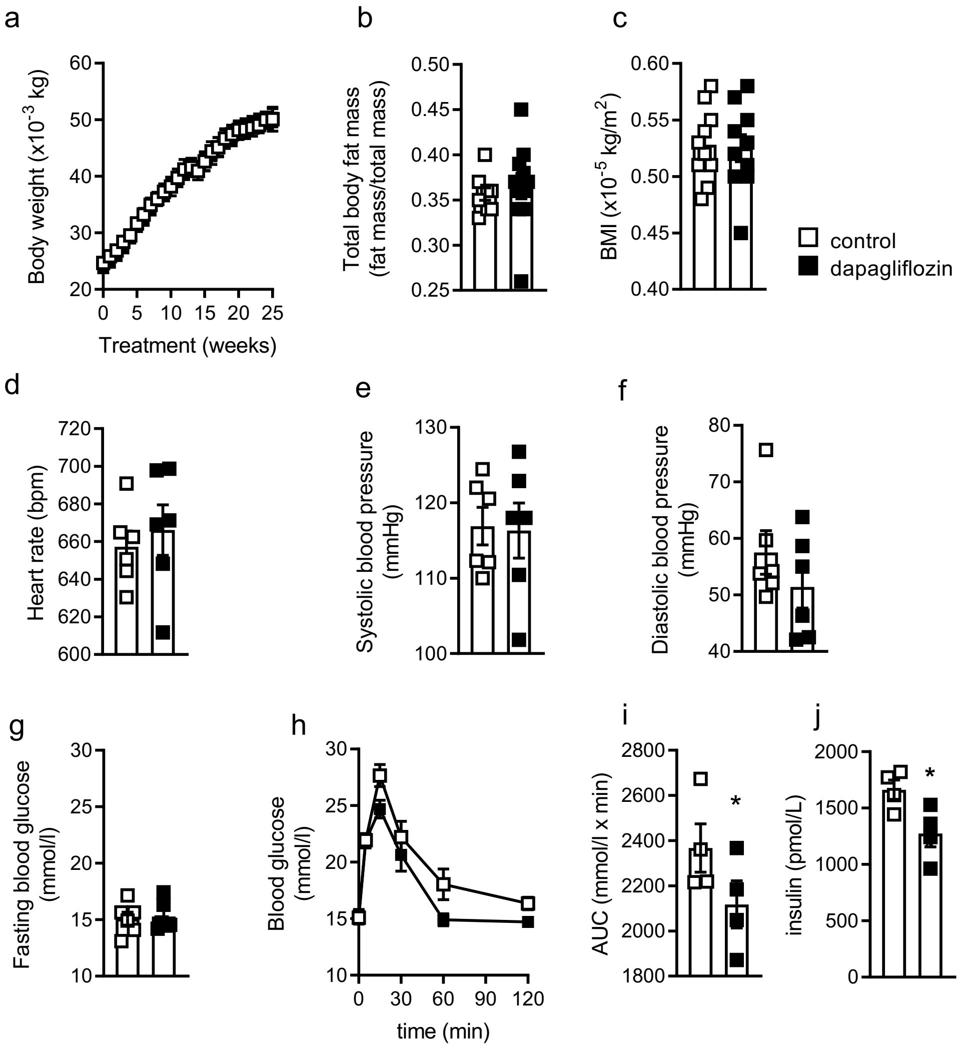

ESM Fig. 1. Feeding with dapagliflozin improves glucose tolerance but has no effect on body weight, blood pressure or heart rate. Male, 8-week-old *Ldlr*$^{-/-}$ mice received diabetogenic diet (DD) supplemented either without (control) or with dapagliflozin (25 mg/kg DD) for 25 weeks. (a) Body weight gain during treatment period; n=10-15, (b) quantification of percentual fat mass related to total body mass; n=10, and (c) body-mass-index (BMI); n=10. (d) Heart rate, (e) systolic and (f) diastolic blood pressure; n=6. (g) Fasting blood glucose, (h) curves of oral glucose tolerance test and (i) the respective area under the curve (AUC) after 8 weeks of treatment; n=9-10; and (j) plasma insulin concentration; n=4. Data are presented as mean ± SEM; (a+e)Two-way ANOVA, (b-g and i-j) Two-tailed unpaired t-test; **P*<0.05 versus control.

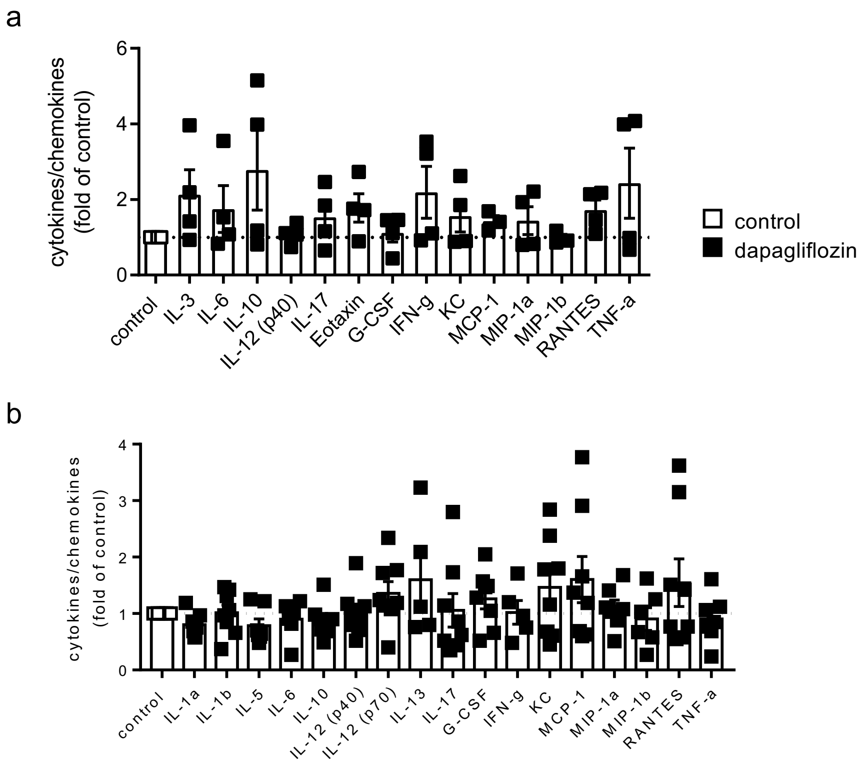

ESM Fig. 2. Chemokine and cytokine secretion is not altered by dapagliflozin treatment. Male, 8-week-old *Ldlr*$^{-/-}$ mice received diabetogenic diet (DD) supplemented either without (control) or with dapagliflozin (25 mg/kg DD). Plasmatic cytokine and chemokine profile after (a) 8 weeks; n=4, or (b), 25 weeks; n=5-8. Data are presented as mean ± SEM, One-way ANOVA.

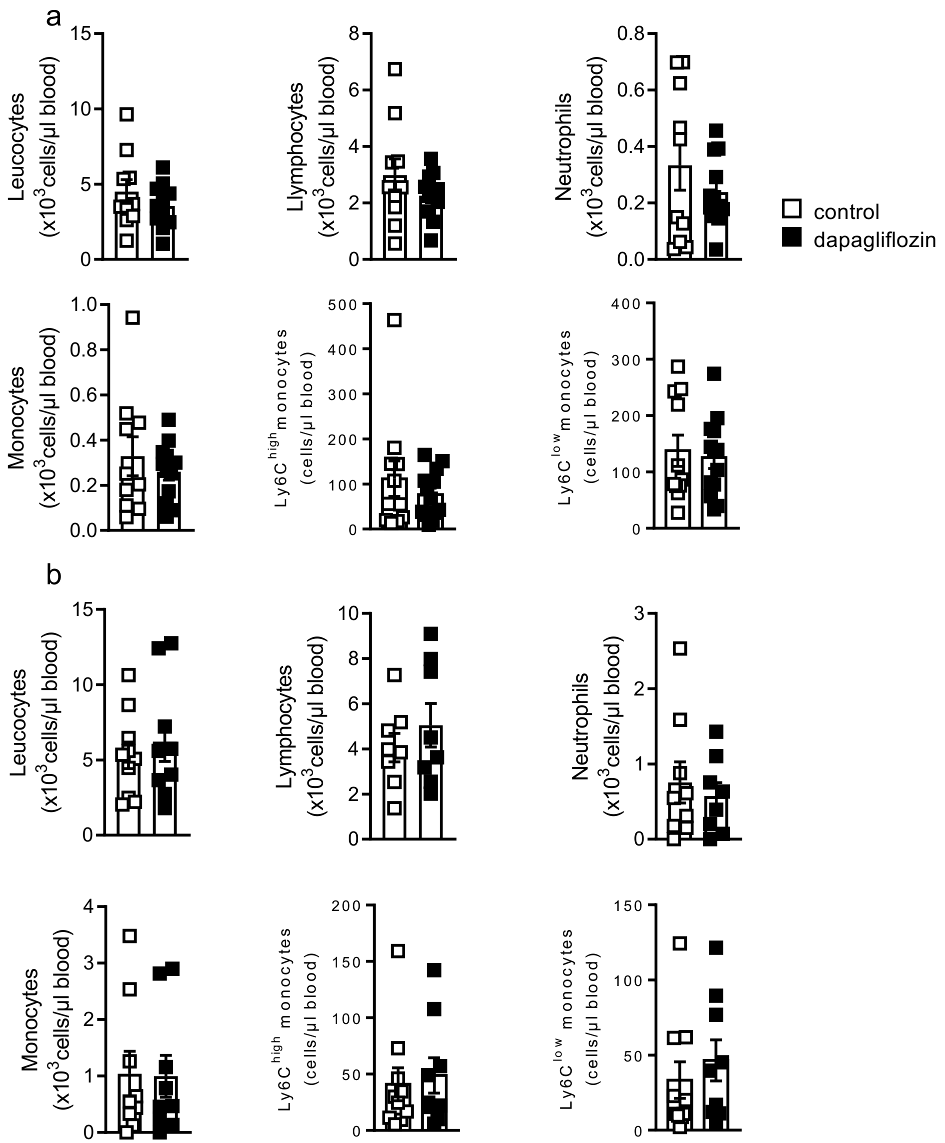

ESM Fig. 3. No effects of dapagliflozin on circulating immune cells. Flow cytometric analysis of leukocytes [$CD45^+$], lymphocytes [$CD45^+CD3^+$/$CD45^+CD19^+$], neutrophils [$CD11b^+Ly6G^+$] and monocytes [$CD11b^+CD115^+$] including $Ly6C^{low}$ and $Ly6C^{high}$ subsets after (a) 8 or (b) 25 weeks of treatment with diabetogenic diet (DD) alone (control) or DD supplemented dapagliflozin (25 mg/kg DD); n=9-13. Data are presented as mean ± SEM, Two-tailed unpaired t-test.

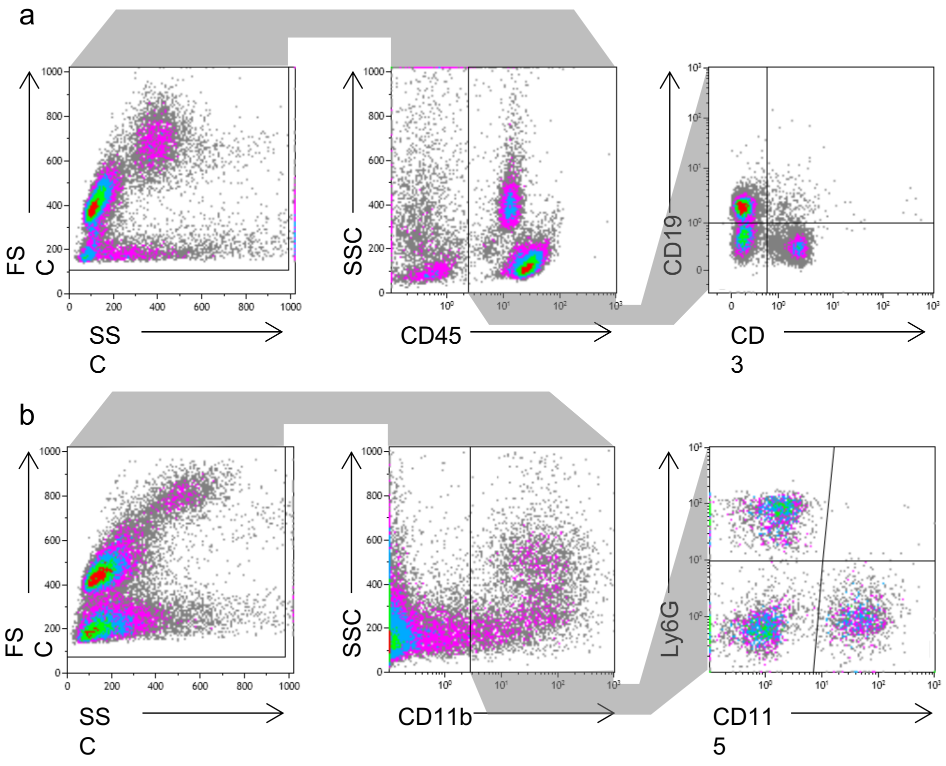

ESM Fig. 4. Gating scheme of circulating immune cells. (a) Representative flow cytometric dot plots and gating strategy for leukocytes [CD45$^+$] and lymphocytes (B cells [CD45$^+$CD19$^+$] and T cells [CD45$^+$CD3$^+$]). (b) Representative flow cytometric plots and gating strategy in blood for neutrophils [CD11b$^+$Ly6G$^+$] and monocytes [CD11b$^+$CD115$^+$].

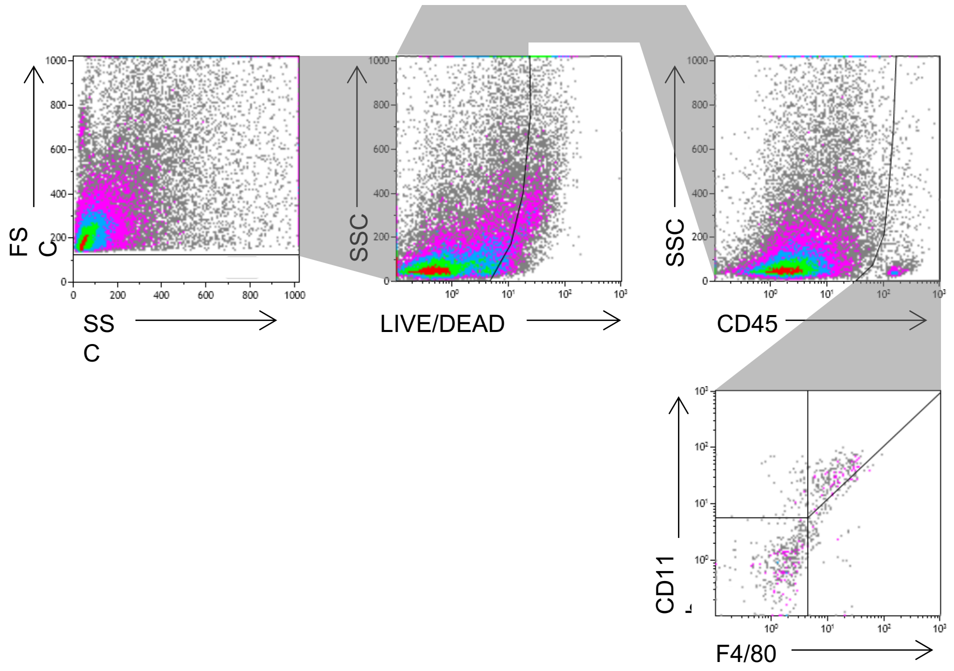

ESM figure 5. Gating scheme of aortic macrophages. Representative flow cytometric dot plots and gating strategy for leukocytes [CD45⁺] and macrophages [CD45⁺CD11b⁺F4/80⁺] in the aorta.

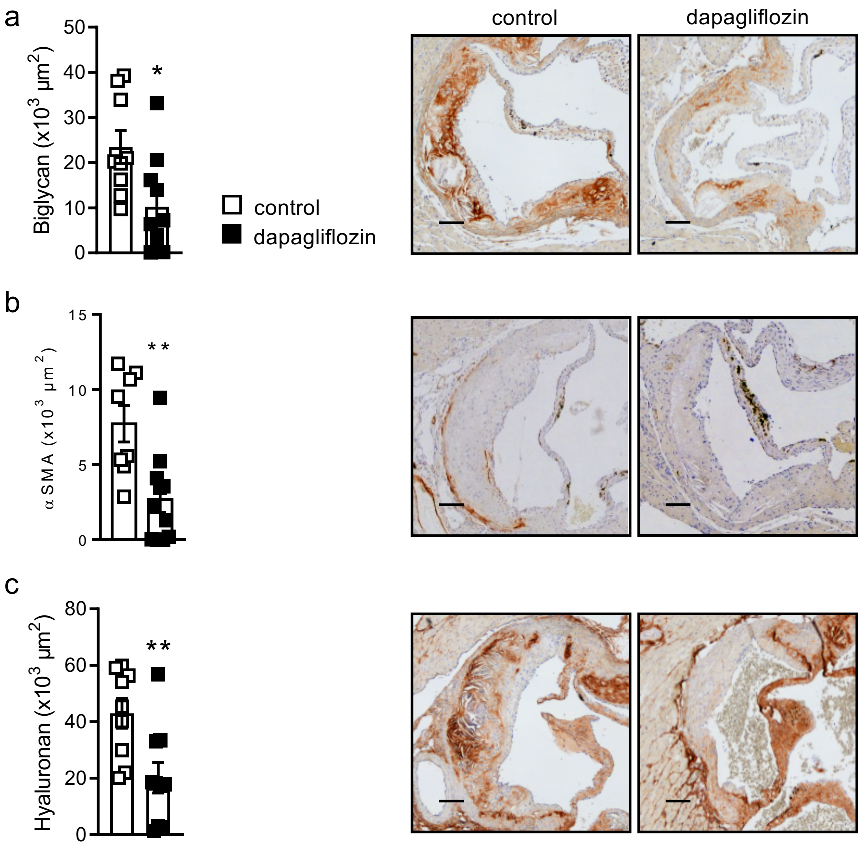

ESM Fig. 6. Dapagliflozin alters plaque composition at the aortic root after 25 weeks of treatment. Male, 8-week-old *Ldlr*$^{-/-}$ mice received diabetogenic diet (DD) supplemented either without (control) or with dapagliflozin (25 mg/kg DD) for 25 weeks. Immunohistochemical staining of (a) biglycan, (b) α-smooth muscle actin (αSMA) and (c) hyaluronan at the aortic root. Respective quantifications and representative pictures are shown; n= 9,12. Scale bars represent 100 µm. Data are presented as mean ± SEM; Two-tailed unpaired t-test; *$P<0.05$, **$P<0.01$.

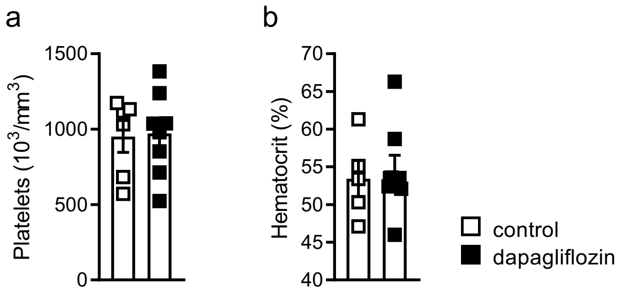

ESM figure 7. Platelet count and hematocrit are not affected by 25 weeks of dapagliflozin treatment in *Ldlr*$^{-/-}$ mice. (a) Platelet count and (b) hematocrit in *Ldlr*$^{-/-}$ mice treated for 25 weeks with diabetogenic diet (DD) supplemented with or without dapagliflozin (25 mg/kg DD); n=6-8. Data are presented as mean ± SEM, Two-tailed unpaired t-test.

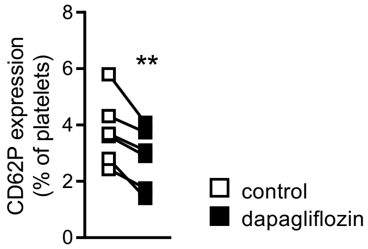

ESM figure 8. Dapagliflozin inhibits CD62P expression in murine platelets *ex vivo*. Platelet CD62P expression before (control) and after incubation of murine whole blood with dapagliflozin (0.5 µM) for 30 min at 37 °C *ex vivo*; n=10. Data are presented as paired values; paired t-test; ***P*<0.01.

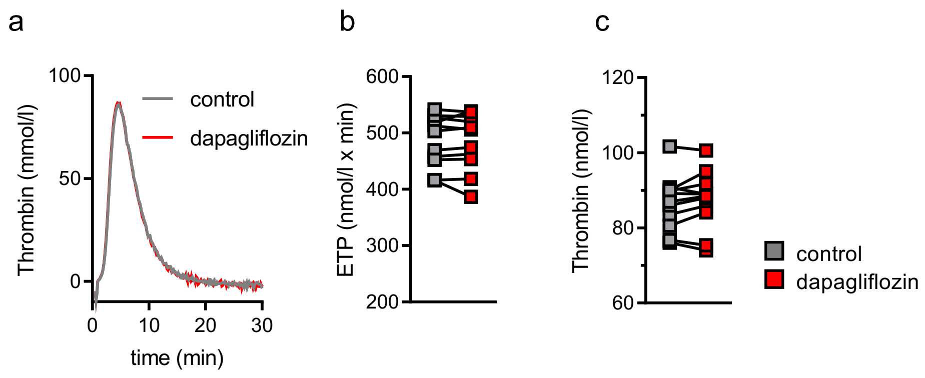

ESM figure 9. Dapagliflozin does not influence thrombin generation in murine plasma *ex vivo*. (a) Endogenous thrombin generation after *ex vivo* incubation of murine platelet poor plasma before (control) and after incubation with dapagliflozin 0.5 μM for 30 min at 37 °C . (b)**,** calculation of the respective area under the curve (endogenous thrombin potential; ETP), and (c) **,** peak height; n=11. Data are presented as paired values: paired t-test.
